# Use of Fluphenazine in Bipolar Disorder With a History of Substance Abuse: A Report of Two Cases

**DOI:** 10.7759/cureus.33556

**Published:** 2023-01-09

**Authors:** Samira Khan, Sira Diakite, Aishwarya Kumar

**Affiliations:** 1 Behavioral Health, West Virginia University Berkeley Medical Center, Martinsburg, USA; 2 Psychiatry and Behavioral Sciences, West Virginia University Berkeley Medical Center, Martinsburg, USA; 3 Psychiatry, West Virginia University Berkeley Medical Center, Martinsburg, USA

**Keywords:** compliance, injectables, anti-psychotics, treatment-resistant, bipolar

## Abstract

One of the major mental health problems with an increased rate of medication failures and treatment resistance arises with the combination of substance use and mood disorders. The purpose of this report is to discuss the off-label use of fluphenazine in managing bipolar disorder in patients with a history of substance abuse, its efficacy, and the importance of having community resources such as assertive community treatment in monitoring treatment progress and improving overall health outcomes. First-generation antipsychotics, like fluphenazine, should be used cautiously with the risks and benefits weighed against possible side effects. This report shows how fluphenazine can successfully be used as an off-label maintenance option in challenging cases.

## Introduction

Treatment of bipolar disorder (BD) can be complex, and it is important to follow up consistently with the affected patients to ensure that they are experiencing adequate control of their symptoms. Unfortunately, recent statistics show that approximately 50% of patients with BD suffer from unresolved depressive morbidity even with standard long-term treatment and follow-up, which can result in disability, cognitive impairment, and excess mortality associated with adverse behavior. The risk of self-harm and suicide is present in patients with BD [1]. The high rates of medication failure and treatment resistance in this population highlight the importance of identifying novel treatment regimens for these patients. Fluphenazine, a first-generation typical antipsychotic (FGA), is indicated for the management of psychosis in patients with schizophrenia disorder. Fluphenazine comes in an oral form, an intramuscular injection for acute symptoms, and a long-acting intramuscular diaconate formula for convenience and to improve medication compliance [2]. Fluphenazine has shown benefits when used off-label for other mental health conditions such as treatment-resistant (TR) BD. There is limited information available when it comes to the use of FGA for TR-BD. Because of the side effect profiles and increased risk for extrapyramidal symptoms (EPS), FGA, such as fluphenazine should be used cautiously with the risks and benefits being weighed against the possible side effects. The two case reports here discuss the benefits of using FGA in managing TR-BD.

## Case presentation

Case 1

The patient, who was a male in his 20s, was brought to the Emergency Room after being found on the streets naked and with bizarre behaviors. The patient was initially uncooperative, belligerent, hypersexual, disorganized, unable to hold a meaningful conversation, and unable to recall the events leading to hospitalization. The patient had a history of being incarcerated for six years due to drug-related charges and was released from jail three months prior to admission. He was released on parole, reported daily, and was living with his parents until he was evicted by his father for sleeping all day, behaving oddly, not responding to people, and having frequent angry outbursts. Due to the patient’s disorganized state, his mother was allotted to be his healthcare surrogate and she provided the admitting team with collateral information. The patient’s mother reported that prior to his incarceration, the patient had a longstanding history of polysubstance abuse and while incarcerated the patient was on psychotropic medications. However, the patient started abusing substances again shortly after being released, which led to his admitting presentation. The initial urine drug screen was negative; however, the patient had reported that his drug of choice was marijuana, and he smoked “till he gets to cloud nine” when he had the means to do so.

On the first day of admission to the unit, the patient was observed to be sexually inappropriate as he pulled down his pants to expose himself to female staff and peers on the unit and masturbated outside his room in front of people. He was a poor historian and lacked insight into his mental illness. Since he was unknown to the team, it was difficult to tease out bipolar I disorder versus schizoaffective disorder versus substance-induced mood disorder versus antisocial personality disorder.

During the admission, he was tried on haloperidol, up to 10mg, and olanzapine, up to 20mg, for mood dysregulation, grandiosity, delusions, and agitation. Both medications were tried for over one week and were discontinued when the patient was observed to be worse on both medications. He was started on carbamazepine 200mg three times a day for bipolar mania with hypersexual behaviors, with olanzapine up to 10mg two times a day for disorganized behaviors. He continued to be disorganized and hypersexual and was observed to be responding to internal stimuli. Olanzapine was tapered and discontinued and fluphenazine was started. The patient showed an improvement with the combination of carbamazepine and fluphenazine as he was more engaged, redirectable, and organized in his thought process. Staff also reported an improvement in him, as he had fewer behavioral outbursts and did not require as-needed medications for agitation and/ or psychosis. An electrocardiogram (EKG) was completed and his QTc was 348, he continued to tolerate his medications well and denied any side effects. The patient was initially started on fluphenazine 5mg orally two times a day, and later the night dose was increased to fluphenazine 10mg orally. In addition, he received fluphenazine 5mg orally as needed consecutively daily for two days for increased agitation and psychosis. An oral dose of fluphenazine 20mg daily is equivalent to 25mg (1ml) of fluphenazine decanoate every three weeks [3]. Hence, he received fluphenazine 25mg Intramuscular long-acting injection per dosing recommendation to improve medication compliance.

Later, carbamazepine was increased to 300mg orally three times a day for mood lability and hypersexuality. The patient showed a significant improvement prior to discharge, as he denied auditory and visual hallucinations, and delusions, and was calm, pleasant, and appropriately social with peers and staff members in the milieu. He was at his baseline according to his mother; he was engaged and was able to hold a meaningful conversation with her on the phone. He did not require any additional fluphenazine by mouth as needed for agitation and psychosis after he got his fluphenazine long-acting injection. He was discharged in a safe and stable condition and was encouraged to continue to take his medications to improve the quality of his life.

Case 2

The patient, who was a female in her 30s, was brought in by her family for concerns about paranoia, perceptual disturbances, and delusions. The patient had a history of BD, substance-induced psychosis, treatment non-compliance, and multiple inpatient admissions with similar presentations. She was disorganized, illogical, with no insight, and believed that “someone wanted to kill me at my house, and there is a guy named Robert who was making me do disgusting things and touching his body.” She was observed to be preoccupied and responded to internal stimuli. The urine drug screen on admission was positive for amphetamines, buprenorphine, and marijuana. A chart review was conducted, and it was noted that the patient was allergic to risperidone, haloperidol, and lurasidone, and tried ziprasidone in the past with no relief. Furthermore, she was last discharged on divalproex 750mg orally two times a day, quetiapine 400mg orally at night for bipolar disorder, and gabapentin 300mg orally three times a day for anxiety and pain. The patient was noncompliant with medications and outpatient follow-up. Historically, this patient had done well with the mood stabilizer of divalproex; therefore, with this admission, a different mood stabilizer was tried.

Prior to resuming her home medications, blood work for hepatic function was within normal range. Divalproex was restarted at 250mg orally twice a day and slowly titrated to up to 875mg orally twice a day; quetiapine was restarted at 100mg by mouth in the morning, quetiapine 400mg by mouth at night for BD, and gabapentin 300 mg orally three times a day for pain and anxiety. Due to her ongoing presentations with agitation, psychosis, and verbal and physical aggression towards those around her, she required frequent administration of olanzapine as needed doses with minimal responses. Quetiapine was initially titrated to address the behaviors; however, the frequency of behaviors increased with each adjustment. Therefore, quetiapine was tapered off and fluphenazine was started and titrated daily. The patient showed significant improvements when she was observed to be more appropriately engaged with full insight, productively participated in group therapy, denied auditory and visual hallucinations, and did not endorse delusions. Although she would have benefited from fluphenazine long-acting injectable to improve medication compliance, the patient was not agreeable. She denied any other adverse side effects and was discharged home in a safe and stable condition. On discharge, her blood test result for hepatic function was within the normal range, valproic acid level was within the normal range at 79.2 ug/mL, and her QTc measurement was 457 ms.

## Discussion

Although intramuscular long-acting fluphenazine has been greatly studied as a treatment for the reduction of positive symptoms in schizophrenia, it may continue to gain traction as a viable off-label maintenance option for TR-BD shown through the successful reduction in the symptoms of the patients as discussed in this case report. Fluphenazine works as an antagonist on dopamine-2 receptors in the mesolimbic, nigrostriatal, and tuberoinfundibular pathways of the brain. Additionally, the drug further antagonizes alpha-1-adrenergic receptors, muscarinic-1 receptors, and histamine-1 receptors, leading to side effects [2]. Dosing criteria for oral, short-acting intramuscular, and long-acting intramuscular injections are detailed in Table 1.

**Table 1:** Fluphenazine Dosing Guidelines Information source: Siragusa et al. [2]

	Oral Tablet	Intramuscular Injection (acute symptoms)	Intramuscular Injection (long-acting)
Dosing range (mg)	2.5-10mg per day; 1.5-2mg (elderly)	1.25-10mg per day	12.5-25mg per day
Dosing Interval	6-8 hours	6-8 hours	28 days
Half-life	14-16 hours	6-10 days	6-10 days

Switching from oral fluphenazine to a long-acting injectable (LAI) formulation is complex due to the variable pharmacokinetics of the drug in the body. The short duration of action as well as the variable absorption rate and peak effect in each patient makes it difficult to convert dosages from oral to LAI fluphenazine. Thus, it is recommended to multiply the daily oral dose by 1.2-1.6 mg or start with 12.5-25 mg for the first dose of intramuscular fluphenazine. The oral and intramuscular doses should be overlapped for about one to two weeks.

While intramuscular fluphenazine may serve as an effective long-term treatment option for patients with refractory bipolar disorder, it is important to make note of the numerous side effects associated with the drug. Both first- and second-generation antipsychotic medications come with significant side effects, some of which are irreversible. Due to a complete blockade of dopamine, FG medications are associated with major side effects, including anticholinergic side effects, sedation, extrapyramidal symptoms, and tardive dyskinesia. Second-generation antipsychotic medications are said to have fewer neuromotor side effects due to the balance between the dopamine and serotonin blockade, but they are associated with elevated risks of dyslipidemia, significant weight gain, metabolic syndrome, and diabetes mellitus [4].

When the use of an FGA is clinically warranted, the treatment algorithm emphasizes the importance of monitoring for these side effects. If a patient experiences tardive dyskinesia or extrapyramidal symptoms, the drug must be switched to a second-generation antipsychotic or discontinued. Neuroleptic malignant syndrome, seizures, liver abnormalities, agranulocytosis, and cardiac abnormalities such as QT elongation and T wave differences are the other rare, but dangerous side effects of fluphenazine use. Patients prescribed this drug must be monitored with an initial and serial EKG and metabolic panels to test for cardiac abnormalities, liver function, and blood counts.

If the side effects experienced by the patient fall into a less severe category, an alternative FGA can be substituted for treatment. These relatively milder side effects are related to the antagonism of the muscarinic, alpha-adrenergic, and histamine receptors and include sedation, dry mouth and eyes, constipation, urinary retention, blurry vision, tachycardia, weight gain, urinary retention, orthostasis, and dizziness. These symptoms can be treated with benztropine, an anti-cholinergic, or sodium benzoate [2].

Extrapyramidal side effects such as dystonia can be prevented by adding an anticholinergic drug to the treatment with FGA. Anticholinergic drugs also come with side effects including dry mouth, constipation, blurred vision, urinary retention, memory impairment, and confusion. For these reasons, there is no strict rule for prophylactic treatment. The decision to start prophylactic anticholinergic treatment before the patient experiences side effects vary according to providers, the patient’s risk factors, and previous side effects with antipsychotic use [5].

Finding alternative pharmacologic interventions in TR-BD is not the only important aspect in the care of these individuals; it is also imperative that they receive proper outpatient follow-up to ensure compliance. Though intramuscular fluphenazine injections could help with this due to their long-acting nature, they still need to be administered in two to four-week intervals, making it difficult for less compliant patients to stay adherent to their medication regimen. Therefore, it is necessary to have community resources available so that patients can be taken care of once they are discharged from an inpatient setting. Assertive community treatment (ACT) is a rehabilitative mental health service for individuals of 18 years and older who have persistent mental illness, frequent inpatient psychiatric hospitalizations, and issues with crisis stabilization. Furthermore, ACT places patients in a community environment to prevent further hospitalizations and promote health maintenance [6]. Literature even shows that ACT remains most effective in populations that experience frequent inpatient hospitalizations [7].

Combining resources into one central location and allowing a variety of mental health-associated professionals to work together through one program allows for more effective and efficient coordination of care for patients with psychiatric disorders. The limitation of this case report is the lack of access to ACT in the state these two patients reside in, which leads to poor follow-up care and eventually nonadherence to treatment. It would be helpful if the patients discussed enrolled in this community-based program to further monitor patient symptoms, treatment, progress, and compliance, which would ensure that fluphenazine maintenance therapy for bipolar disorder remains effective.

## Conclusions

The challenge of treating individuals with mood and substance disorders may be discouraging at times but the knowledge provided by this case report may give providers hope. Fluphenazine was successfully used in both oral and injectable forms in individuals with treatment-resistant bipolar disorder and a history of substance abuse. In the first case, a male in his 20s was newly diagnosed with mood disorder, had failed multiple antipsychotics, and continued to worsen in mood and behaviors until he was started on fluphenazine with a mood stabilizer, carbamazepine. Additionally, in the second case, a female in her 30s, with chronic mental health concerns and non-compliance on an outpatient basis, too responded well to fluphenazine and the mood stabilizer, valproic acid. Using antipsychotics and mood stabilizers continues to show benefits in treating challenging mental health concerns that may or may not be comorbid with substance abuse.
